# Supplementary material for: Proteome-Wide Screening of Potential Vaccine Targets against Brucella melitensis
Source: Vaccines (Basel). 2023 Jan 25;11(2):263. doi: 10.3390/vaccines11020263 (PMC9966016; doi:10.3390/vaccines11020263)
Supplement: Supplementary file 1 [file vaccines-11-00263-s001.zip › vaccines-2084278-supplementary.pdf]

Table S1. Predicted T-cells epitopes.

MHC I	Percentile score	MHC-II	Percentile score
TAGERGPIL	0.48	TAGERGPIL	0.48
GAPIPDNQN	35		
		GAPIPDNQN	35
HPRTHLRSAT KR	0		
	0	HPRTHLRSAT	0
		KR	0
WTNAEAEQV	0		
		WTNAEAEQV	0
RTRESTQGH	0.06		
RLGTHYESI	0.5		
		RLGTHYESI	0.5
QPKCPVHHY	0.1		
		QPKCPVHHY	0.1
KTGNPDAYY	0.03		
		KTGNPDAYY	0.03
NSFNGPVEQ	2.7		
		NSFNGPVEQ	2.7
KEPPLCISG	4.4		
		KEPPLCISG	4.4
NTIALNEKI	0.05		
		NTIALNEKI	0.05
YQGDDYAKV	0.08		
		YQGDDYAKV	0.08
EPDYEAVNA	2.2		
		EPDYEAVNA	2.2
EAEAKAEAEK	0.56		
		EAEAKAEAEK	0.56
SIGNHGQPI	2.7		
		SIGNHGQPI	2.7
AIGREGNSAK	0.34		
		AIGREGNSAK	0.34
SSDDFTQSA	0.28		
		SSDDFTQSA	0.28
EADAYYASR	0.3		
		EADAYYASR	0.3
RPLESRFALE	0.21		
		RPLESRFALE	0.21
PYDYDALAPF	0.29		
		PYDYDALAPF	0.29
GLEGKSLEEI	2.6		
		GLEGKSLEEI	2.6
KGFDAARVG	8.7		
		KGFDAARVG	8.7
NYPDLPAEFS	0.55		
		NYPDLPAEFS	0.55
GEKDGKIVPA	1.5		
		GEKDGKIVPA	1.5
NTHHHLGPE	11		
		NTHHHLGPE	11
NYSDKPEPL	0.25		
		NYSDKPEPL	0.25
RERRLAQAM	0.4		

		RERRLAQAM	0.4
YPSAVAGGA	1.6	YPSAVAGGA	1.6
LAGNIKQAR	0.61	LAGNIKQAR	0.61
KAGIEGAAKK	0.48	KAGIEGAAKK	0.48
FAGLDLGSLL	1.7	FAGLDLGSLL	1.7
SGAATGTTP	16	SGAATGTTP	16
TTGATVDLSG	20	TTGATVDLSG	20
KINDSFRTF	0.01	KINDSFRTF	0.01
NQIAPQQPV	0.27	NQIAPQQPV	0.27
LPQQAQPQGV	0.36	LPQQAQPQGV	0.36
SLDPSIGMA	0.5	SLDPSIGMA	0.5
YAPEPQPQT	1.2	YAPEPQPQT	1.2
PEAKISDAL	0.28	PEAKISDAL	0.28
APQGQAPQQV	0.36	APQGQAPQQV	0.36
DTSGYFPAA	0.14	DTSGYFPAA	0.14
PSADSAPIM	4.9	PSADSAPIM	4.9
VLRTDKRSG	8.3	VLRTDKRSG	8.3
ALRAKPQTEY	0.1	ALRAKPQTEY	0.1
EPIYSAGQK	2.3	EPIYSAGQK	2.3
FADYQQAMKK	1.1	FADYQQAMKK	1.1
AGASSTNVSV	2.9	AGASSTNVSV	2.9
KFYEALDKK	0.22	KFYEALDKK	0.22
FADYQQAMKK	1.1	FADYQQAMKK	1.1
FAVSQGFKG	10	FAVSQGFKG	10
GASSTNVSV	1.2	GASSTNVSV	1.2
KFYEALDKK	0.22	KFYEALDKK	0.22
VPQQRQPGS	1.9	VPQQRQPGS	1.9
IQMGDIADQV	0.18	IQMGDIADQV	0.18
REISAAEGR	2	REISAAEGR	2
RIKNLKNPL	0.3		

		RIKNLKNPL	0.3
RTSTKDKTP	4.7		
		RTSTKDKTP	4.7
KSGVSGNRLR	0.64		
		KSGVSGNRLR	0.64
VSRNISGAK	0.05		
		VSRNISGAK	0.05
SALYSADNY	0.25		
		SALYSADNY	0.25
RMGRDVSDY	0.14		
		RMGRDVSDY	0.14
TVDGVPLSH	0.21		
		TVDGVPLSH	0.21
DTFDQPFGT	0.12		
		DTFDQPFGT	0.12
AVLTPGVAG	5		
		AVLTPGVAG	5
MVFDAAAGI	0.04		
		MVFDAAAGI	0.04
RLRALNEAF	0.02		
		RLRALNEAF	0.02
YNVQGAAGF	1.1		
		YNVQGAAGF	1.1
LLRDGGANGA	1.3		
		LLRDGGANGA	1.3
SSSSLIGYS	6.9		
		SSSSLIGYS	6.9
ESDQTGSSP	2.4		
		ESDQTGSSP	2.4
FSWSGSPAI	0.15		
		FSWSGSPAI	0.15
PGAGLSAGI	15		
		PGAGLSAGI	15
GTIEVSVPF	0.5		
		GTIEVSVPF	0.5
RQKANSEFTY	0.01		
		RQKANSEFTY	0.01
FALSNATGV	0.36		
		FALSNATGV	0.36
LPNNAGDLG	3.8		
		LPNNAGDLG	3.8
YGNERPVAV	0.5		
		YGNERPVAV	0.5
SPVGSNTTN	3.1		
		SPVGSNTTN	3.1
ASNSTSAANK	0.5		
		ASNSTSAANK	0.5
PTQPMDPTQY	0.1		

Table S2. Top 20 docked complexes of vaccine and MHC-I molecules.

Solution No	Score	Area	ACE	Transformation
1	20052	3064.00	430.80	0.93 -1.33 -2.46 49.25 29.79 62.95
2	19230	3874.50	-257.82	0.31 0.49 -1.73 23.00 53.50 -11.09
3	18770	3598.30	382.90	1.37 -0.94 -1.86 49.31 35.18 61.85
4	18698	3819.60	69.98	-0.80 -0.91 -0.78 16.60 31.64 55.26
5	18612	3093.80	62.86	-0.66 -0.43 -2.33 38.78 73.53 57.88
6	18404	2991.40	442.40	1.35 -0.99 -2.11 49.66 32.24 64.65
7	18264	2826.80	398.82	-2.51 0.20 0.20 18.94 15.31 62.91
8	18212	2739.90	141.14	-1.51 -0.40 -2.39 33.91 52.55 54.26
9	18180	2790.80	-138.43	1.78 -0.74 -0.64 27.33 50.96 59.02
10	17952	2448.00	-82.03	-1.06 -0.12 -2.18 30.06 52.91 42.25
11	17784	2886.90	349.38	-0.34 -0.41 -0.48 9.67 42.60 42.05
12	17546	2405.50	232.28	0.66 -1.09 -2.75 55.04 19.22 63.68
13	17532	2373.30	458.52	3.04 0.06 0.18 -4.58 12.57 80.70
14	17338	2580.20	-91.78	-2.35 -0.06 -1.84 19.51 49.41 60.39
15	17330	3714.80	-607.82	-2.31 -0.17 2.46 53.45 20.34 64.32
16	17262	2569.50	354.21	2.16 -1.05 2.45 51.45 31.98 66.97
17	17240	3145.40	-416.03	-0.30 0.57 -0.21 12.13 6.29 7.78
18	17160	2216.30	287.15	0.25 1.28 -2.87 39.86 52.21 -33.12
19	17126	2802.30	-237.38	-0.31 0.43 -0.52 -0.27 21.72 13.74
20	17096	3403.10	-12.54	-1.11 -0.25 -2.19 29.97 54.19 41.64

Table S3. Top 20 docked complexes of vaccine and MHC-II molecule.

Solution No	Score	Area	ACE	Transformation
1	19926	2989.00	285.99	-1.97 0.63 -1.24 45.78 107.07 0.60
2	18652	3252.80	226.24	-1.72 -0.02 0.46 80.79 62.03 13.19
3	18474	3020.70	157.92	-2.79 0.59 2.34 130.56 91.47 24.80
4	18264	2923.20	-228.22	-0.20 -0.20 1.21 137.38 10.49 -0.96
5	18122	3186.60	-143.48	-0.26 0.29 2.05 97.13 91.20 -30.28
6	18062	2391.00	192.51	1.26 0.78 -0.01 106.36 109.37 -25.36
7	18046	2445.90	296.33	0.23 0.83 -2.56 145.40 52.70 -16.12
8	17902	2680.40	211.19	-0.30 0.31 1.87 96.97 93.36 -30.28
9	17608	2596.10	381.93	-1.31 0.85 -2.94 111.63 121.49 -13.16
10	17518	2958.80	348.72	-0.49 0.99 1.63 150.86 54.34 -17.96
11	17402	2661.90	154.01	-1.81 0.31 2.74 142.39 75.21 13.31
12	17354	2660.80	104.17	-1.65 0.10 -2.62 136.27 85.29 12.13
13	17096	2737.90	269.33	-2.05 -0.02 0.83 91.84 67.10 30.38
14	16934	2506.60	-18.83	-0.76 0.33 -0.48 76.52 72.10 -14.73
15	16902	2626.00	370.38	2.96 -0.99 0.55 126.89 33.33 34.50
16	16820	2942.60	319.56	-1.74 -1.43 1.11 103.66 50.42 27.27
17	16694	2712.00	346.10	1.99 0.14 2.40 105.32 24.37 21.00
18	16638	2811.10	220.07	-1.98 -0.92 1.99 146.64 29.96 22.84
19	16624	3217.50	-135.88	-0.36 0.29 -0.05 107.30 37.68 -39.42
20	16602	2549.00	144.91	-1.21 -0.83 -1.18 56.14 117.09 57.53

Table S4. Top 20 docked complexes of vaccine and TLR-4molecule.

Solution No	Score	Area	ACE	Transformation
1	21596	4181.90	429.96	0.42 -0.64 1.98 21.60 -32.60 -64.27
2	20322	2657.20	427.08	-2.77 0.01 -2.15 19.53 -3.77 4.00
3	19008	2850.00	243.89	-2.78 -0.31 2.18 -51.77 1.44 18.63
4	18718	3337.40	-5.05	-0.79 -1.04 1.18 -32.42 10.68 -17.69
5	18654	3630.70	-56.43	2.97 0.44 0.95 -59.97 -12.14 -55.61
6	18620	2919.30	124.39	2.95 -0.56 1.23 -35.51 -47.42 -7.00
7	18310	3538.20	139.79	-1.59 0.20 -1.28 -100.38 38.63 -37.00
8	18192	3104.30	220.58	0.56 -0.42 0.21 -66.83 -1.24 2.40
9	18184	4290.30	-1.57	-2.03 -0.60 2.39 -19.13 31.64 -15.47
10	18116	2633.00	315.92	3.12 -0.55 1.26 -34.35 -46.36 -13.95
11	18000	2857.20	499.22	2.49 1.05 0.16 -52.36 -30.79 -59.05
12	17958	2551.00	460.57	0.03 0.45 -0.63 -50.78 -31.04 -58.69
13	17920	2595.90	206.79	2.41 -0.11 0.71 -15.51 12.03 -47.59
14	17906	2384.50	210.66	2.76 0.70 1.22 -50.26 -14.56 -69.48
15	17690	2522.70	33.22	-0.03 1.02 1.55 -14.48 53.14 -76.81
16	17640	2749.10	54.31	-0.44 1.08 1.10 52.56 7.16 -75.19
17	17612	3080.70	79.52	0.25 0.77 2.41 2.75 10.31 -51.04
18	17554	3256.50	42.58	0.78 0.76 2.78 -57.00 -8.98 -49.23
19	17496	2674.80	291.40	-0.26 0.59 -0.70 -53.94 -38.49 -55.57
20	17468	3466.30	75.91	2.43 -0.04 -2.52 -36.11 24.77 1.37
